# Standardising Resident Doctor Handbooks Across East and Central Lancashire Localities

**DOI:** 10.1192/bjo.2026.11413

**Published:** 2026-06-30

**Authors:** Mihir Sunil Kolhe, Deoman Gurung, Thuya Han

**Affiliations:** LCSFT, Preston, United Kingdom

## Abstract

**Aims:**

Resident doctor handbooks are a key system-level resource supporting safe, effective working and trainee induction. Within Lancashire and South Cumbria NHS Foundation Trust (LSCFT), variation was identified between the East and Central psychiatry locality handbooks, reflecting differences in site coverage and resulting in gaps in essential information. This inconsistency risked inefficiency, uncertainty, and increased anxiety for new starters. The aim of this quality improvement project was to improve the consistency, accessibility, and reliability of resident doctor information by identifying and addressing variation in handbook content and standardising guidance across the East and Central localities.

**Methods:**

A baseline comparison of the East and Central resident doctor handbooks was undertaken to identify missing content, inconsistencies, and areas lacking sufficient detail. Content was reviewed against key trainee domains, including emergency management, risk and legal processes, physical health responsibilities, and routine ward duties. Identified gaps informed a structured programme of handbook revision. Iterative updates were discussed with the Central locality educational lead and reviewed with the consultant body at consultant meetings and within monthly college tutor meetings. Finalised content was reviewed with the East locality educational lead to validate accuracy and ensure parity across both handbooks.

**Results:**

The intervention resulted in a standardised handbook structure and improved content parity across the East and Central localities. Previously missing sections were added to the Central handbook, new clinically relevant sections were introduced to both handbooks, and existing sections were expanded to improve clarity and usability. Updates focused on psychiatric and medical emergencies, management of acute disturbance, risk and legalprocesses, physical health assessment, and practical ward guidance. Positive feedback was received from educational leads in both localities and from the consultant body, alongside informal positive trainee feedback regarding clarity and usability. The revised handbooks were distributed to new starters in August 2025, with planned redistribution to subsequent cohorts in February 2026.

**Conclusion:**

This quality improvement project successfully addressed unwarranted variation in resident doctor handbook content across two psychiatry localities through standardisation and educational lead(s) co-production. Improving the consistency and accessibility of trainee information supports safer working, enhances trainee experience, and reduces uncertainty for new starters. Future work will include extending the intervention to the North locality, incorporating additional clinically relevant content such as venous thromboembolism assessment guidance, and ensuring parity across specialty-specific handbooks, including learning disability and forensic services.

